# 1-Phenylethane-1,2-diyl 1,1′-biphenyl-2,2′-dicarboxylate

**DOI:** 10.1107/S1600536812018065

**Published:** 2012-04-28

**Authors:** Hoong-Kun Fun, Ching Kheng Quah, Dongdong Wu

**Affiliations:** a X-ray Crystallography Unit, School of Physics, Universiti Sains Malaysia, 11800 USM, Penang, Malaysia; b School of Chemistry and Chemical Engineering, Nanjing University, Nanjing, 210093, People’s Republic of China

## Abstract

In the title compound, C_22_H_16_O_4_, the 7-phenyl ring is inclined at dihedral angles of 36.73 (9) and 69.37 (9)° with respect to the biphenyl benzene rings. The two benzene rings of the biphenyl unit form a dihedral angle of 55.99 (8)°. There are no significant hydrogen bonds observed in the crystal of this compound.

## Related literature

For a related structure, references to other similar structures and chemical and biological background, see: Fun *et al.* (2012) ▶. For the preparation, see: Wu *et al.* (2012) ▶.
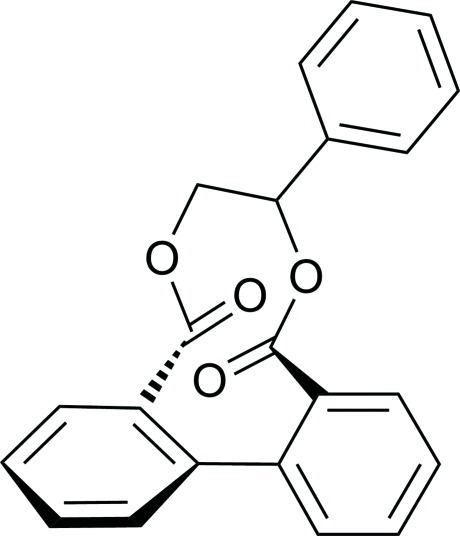


## Experimental

### 

#### Crystal data

C_22_H_16_O_4_
*M* *_r_* = 344.35
Orthorhombic, 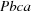

*a* = 21.2454 (15) Å
*b* = 13.2363 (7) Å
*c* = 12.1954 (7) Å
*V* = 3429.5 (4) Å^3^
*Z* = 8
Mo *K*α radiation
μ = 0.09 mm^−1^
*T* = 296 K
0.27 × 0.22 × 0.08 mm

#### Data collection

Bruker SMART APEXII DUO CCD diffractometer
Absorption correction: multi-scan (*SADABS*; Bruker, 2009 ▶) *T*_min_ = 0.976, *T*_max_ = 0.993
67362 measured reflections
5014 independent reflections
2961 reflections with *I* > 2σ(*I*)
*R*_int_ = 0.075

#### Refinement

*R*[*F* ^2^ > 2σ(*F* ^2^)] = 0.044
*wR*(*F* ^2^) = 0.113
*S* = 0.99
5014 reflections
235 parameters
H-atom parameters constrained
Δρ_max_ = 0.16 e Å^−3^
Δρ_min_ = −0.16 e Å^−3^

### 

Data collection: *APEX2* (Bruker, 2009 ▶); cell refinement: *SAINT* (Bruker, 2009 ▶); data reduction: *SAINT*; program(s) used to solve structure: *SHELXTL* (Sheldrick, 2008 ▶); program(s) used to refine structure: *SHELXTL*; molecular graphics: *SHELXTL*; software used to prepare material for publication: *SHELXTL* and *PLATON* (Spek, 2009 ▶).

## Supplementary Material

Crystal structure: contains datablock(s) global, I. DOI: 10.1107/S1600536812018065/hb6748sup1.cif

Structure factors: contains datablock(s) I. DOI: 10.1107/S1600536812018065/hb6748Isup2.hkl

Supplementary material file. DOI: 10.1107/S1600536812018065/hb6748Isup3.cml

Additional supplementary materials:  crystallographic information; 3D view; checkCIF report

## Figures and Tables

**Fig. 1.**
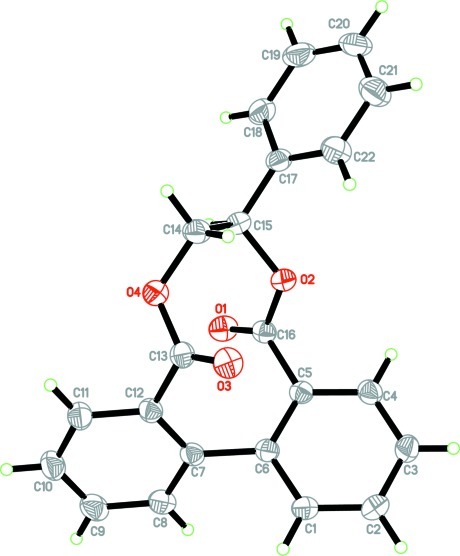
The molecular structure of the title compound showing 30% probability displacement ellipsoids for non-H atoms.

## supplementary crystallographic information

### Comment

As part of our ongoing synthetic and structural studies of bis-lactones containing a biaryl motif (Wu *et al.*, 2012; Fun *et al.* 2012), we now report the structure of the title compound, (I).

In the title compound, Fig. 1, the terminal benzene ring (C17-C22) inclines at dihedral angles of 36.73 (9) and 69.37 (9)° with the other two benzene rings (C1-C6 and C7-C12), respectively. The two benzene rings form a dihedral angle of 55.99 (8)°.

### Experimental

The title compound was the major diastereoisomer of the photoreaction products of 9,10-phenanthrenedione with styrene via a photocycloaddition-photooxidation sequence. The compound was purified by flash column chromatography with ethyl acetate/petroleum ether (1:9) as eluents. Colourless plates of the title compound was obtained from slow evaporation of an acetone and petroleum ether solution (1:10).

### Refinement

All H atoms were positioned geometrically and refined using a riding model with C–H = 0.93-0.98 Å and *U*_iso_(H) = 1.2 *U*_eq_(C).

### Crystal data

**Table:**

C_22_H_16_O_4_	*F*(000) = 1440
*M**_r_* = 344.35	*D*_x_ = 1.334 Mg m^−^^3^
Orthorhombic, *P**b**c**a*	Mo *K*α radiation, λ = 0.71073 Å
Hall symbol: -P 2ac 2ab	Cell parameters from 6139 reflections
*a* = 21.2454 (15) Å	θ = 2.5–24.0°
*b* = 13.2363 (7) Å	µ = 0.09 mm^−^^1^
*c* = 12.1954 (7) Å	*T* = 296 K
*V* = 3429.5 (4) Å^3^	Plate, colourless
*Z* = 8	0.27 × 0.22 × 0.08 mm

### Data collection

**Table:**

Bruker SMART APEXII DUO CCD diffractometer	5014 independent reflections
Radiation source: fine-focus sealed tube	2961 reflections with *I* > 2σ(*I*)
Graphite monochromator	*R*_int_ = 0.075
φ and ω scans	θ_max_ = 30.0°, θ_min_ = 1.9°
Absorption correction: multi-scan (*SADABS*; Bruker, 2009)	*h* = −29→29
*T*_min_ = 0.976, *T*_max_ = 0.993	*k* = −18→18
67362 measured reflections	*l* = −17→17

### Refinement

**Table:**

Refinement on *F*^2^	Primary atom site location: structure-invariant direct methods
Least-squares matrix: full	Secondary atom site location: difference Fourier map
*R*[*F*^2^ > 2σ(*F*^2^)] = 0.044	Hydrogen site location: inferred from neighbouring sites
*wR*(*F*^2^) = 0.113	H-atom parameters constrained
*S* = 0.99	*w* = 1/[σ^2^(*F*_o_^2^) + (0.0382*P*)^2^ + 0.8815*P*] where *P* = (*F*_o_^2^ + 2*F*_c_^2^)/3
5014 reflections	(Δ/σ)_max_ = 0.001
235 parameters	Δρ_max_ = 0.16 e Å^−^^3^
0 restraints	Δρ_min_ = −0.16 e Å^−^^3^

### Special details

**Table:**

Geometry. All esds (except the esd in the dihedral angle between two l.s. planes) are estimated using the full covariance matrix. The cell esds are taken into account individually in the estimation of esds in distances, angles and torsion angles; correlations between esds in cell parameters are only used when they are defined by crystal symmetry. An approximate (isotropic) treatment of cell esds is used for estimating esds involving l.s. planes.
Refinement. Refinement of F^2^ against ALL reflections. The weighted R-factor wR and goodness of fit S are based on F^2^, conventional R-factors R are based on F, with F set to zero for negative F^2^. The threshold expression of F^2^ > 2sigma(F^2^) is used only for calculating R-factors(gt) etc. and is not relevant to the choice of reflections for refinement. R-factors based on F^2^ are statistically about twice as large as those based on F, and R- factors based on ALL data will be even larger.

### Fractional atomic coordinates and isotropic or equivalent isotropic displacement parameters (Å^2^)

**Table:**

	*x*	*y*	*z*	*U*_iso_*/*U*_eq_	
O1	0.26894 (5)	0.00729 (8)	−0.06424 (9)	0.0525 (3)	
O2	0.28774 (4)	0.14794 (8)	0.03621 (8)	0.0431 (2)	
O3	0.18085 (5)	0.27892 (8)	−0.05404 (9)	0.0517 (3)	
O4	0.25786 (5)	0.22593 (9)	−0.16930 (8)	0.0494 (3)	
C1	0.07994 (7)	0.07069 (12)	0.06878 (12)	0.0481 (4)	
H1A	0.0415	0.0732	0.0323	0.058*	
C2	0.08085 (7)	0.06903 (14)	0.18225 (13)	0.0551 (4)	
H2A	0.0432	0.0708	0.2211	0.066*	
C3	0.13733 (8)	0.06473 (13)	0.23800 (13)	0.0525 (4)	
H3A	0.1378	0.0628	0.3142	0.063*	
C4	0.19297 (7)	0.06329 (12)	0.18020 (12)	0.0461 (4)	
H4A	0.2311	0.0611	0.2177	0.055*	
C5	0.19263 (6)	0.06515 (10)	0.06611 (11)	0.0375 (3)	
C6	0.13535 (6)	0.06867 (11)	0.00836 (11)	0.0382 (3)	
C7	0.13142 (6)	0.07071 (11)	−0.11413 (11)	0.0397 (3)	
C8	0.09627 (7)	−0.00380 (13)	−0.16669 (14)	0.0492 (4)	
H8A	0.0757	−0.0524	−0.1250	0.059*	
C9	0.09149 (8)	−0.00670 (14)	−0.27979 (15)	0.0587 (5)	
H9A	0.0677	−0.0569	−0.3134	0.070*	
C10	0.12184 (8)	0.06460 (15)	−0.34267 (14)	0.0602 (5)	
H10A	0.1185	0.0625	−0.4187	0.072*	
C11	0.15715 (7)	0.13905 (14)	−0.29305 (12)	0.0511 (4)	
H11A	0.1781	0.1866	−0.3357	0.061*	
C12	0.16148 (6)	0.14330 (11)	−0.17924 (11)	0.0404 (3)	
C13	0.19893 (7)	0.22471 (12)	−0.12604 (12)	0.0417 (3)	
C14	0.30724 (7)	0.26949 (13)	−0.10300 (13)	0.0508 (4)	
H14A	0.2900	0.3201	−0.0539	0.061*	
H14B	0.3386	0.3014	−0.1493	0.061*	
C15	0.33672 (6)	0.18389 (12)	−0.03764 (12)	0.0433 (3)	
H15A	0.3492	0.1293	−0.0874	0.052*	
C16	0.25324 (6)	0.06640 (11)	0.00485 (12)	0.0384 (3)	
C17	0.39237 (6)	0.21678 (12)	0.02917 (12)	0.0446 (3)	
C18	0.45246 (7)	0.18547 (15)	0.00219 (14)	0.0592 (4)	
H18A	0.4587	0.1439	−0.0583	0.071*	
C19	0.50365 (8)	0.21590 (18)	0.06533 (17)	0.0737 (6)	
H19A	0.5441	0.1957	0.0460	0.088*	
C20	0.49468 (9)	0.27522 (16)	0.15542 (18)	0.0722 (6)	
H20A	0.5288	0.2936	0.1989	0.087*	
C21	0.43562 (10)	0.30752 (16)	0.18154 (16)	0.0706 (5)	
H21A	0.4298	0.3491	0.2422	0.085*	
C22	0.38446 (8)	0.27928 (14)	0.11904 (14)	0.0584 (4)	
H22A	0.3445	0.3024	0.1374	0.070*	

### Atomic displacement parameters (Å^2^)

**Table:**

	*U*^11^	*U*^22^	*U*^33^	*U*^12^	*U*^13^	*U*^23^
O1	0.0485 (6)	0.0438 (6)	0.0653 (7)	0.0023 (5)	0.0063 (5)	−0.0075 (5)
O2	0.0387 (5)	0.0468 (6)	0.0439 (5)	−0.0077 (4)	0.0023 (4)	0.0004 (5)
O3	0.0564 (6)	0.0452 (6)	0.0536 (6)	0.0025 (5)	−0.0006 (5)	−0.0029 (5)
O4	0.0434 (5)	0.0608 (7)	0.0440 (6)	−0.0064 (5)	−0.0021 (4)	0.0075 (5)
C1	0.0363 (7)	0.0583 (10)	0.0498 (9)	−0.0033 (7)	−0.0002 (6)	0.0030 (8)
C2	0.0455 (8)	0.0677 (12)	0.0521 (10)	−0.0073 (8)	0.0113 (7)	0.0018 (8)
C3	0.0584 (9)	0.0594 (10)	0.0398 (8)	−0.0107 (8)	0.0029 (7)	0.0032 (7)
C4	0.0472 (8)	0.0479 (9)	0.0432 (8)	−0.0070 (7)	−0.0058 (6)	0.0062 (7)
C5	0.0382 (7)	0.0333 (7)	0.0410 (7)	−0.0027 (6)	−0.0011 (6)	0.0038 (6)
C6	0.0365 (7)	0.0378 (7)	0.0405 (7)	−0.0013 (6)	−0.0019 (6)	0.0027 (6)
C7	0.0333 (6)	0.0443 (8)	0.0416 (7)	0.0052 (6)	−0.0023 (6)	−0.0020 (6)
C8	0.0420 (7)	0.0500 (9)	0.0557 (9)	0.0000 (7)	−0.0016 (7)	−0.0073 (8)
C9	0.0511 (9)	0.0685 (12)	0.0566 (10)	0.0000 (9)	−0.0052 (8)	−0.0228 (9)
C10	0.0563 (9)	0.0838 (14)	0.0405 (8)	0.0066 (10)	−0.0045 (7)	−0.0124 (9)
C11	0.0483 (8)	0.0649 (11)	0.0400 (8)	0.0051 (8)	−0.0009 (7)	0.0021 (8)
C12	0.0361 (6)	0.0463 (8)	0.0387 (7)	0.0056 (6)	−0.0028 (6)	0.0005 (6)
C13	0.0427 (7)	0.0434 (8)	0.0391 (7)	0.0019 (6)	−0.0035 (6)	0.0107 (7)
C14	0.0436 (8)	0.0549 (10)	0.0539 (9)	−0.0118 (7)	−0.0041 (7)	0.0081 (8)
C15	0.0376 (7)	0.0486 (8)	0.0437 (8)	−0.0068 (6)	0.0042 (6)	0.0024 (7)
C16	0.0365 (6)	0.0354 (7)	0.0433 (7)	0.0013 (6)	−0.0053 (6)	0.0055 (6)
C17	0.0389 (7)	0.0483 (9)	0.0467 (8)	−0.0072 (7)	0.0008 (6)	0.0089 (7)
C18	0.0435 (8)	0.0797 (13)	0.0544 (10)	−0.0010 (8)	0.0062 (7)	0.0063 (9)
C19	0.0367 (8)	0.1048 (17)	0.0796 (14)	−0.0100 (10)	0.0000 (9)	0.0220 (13)
C20	0.0571 (10)	0.0842 (14)	0.0752 (13)	−0.0311 (10)	−0.0176 (10)	0.0184 (11)
C21	0.0758 (12)	0.0676 (13)	0.0682 (12)	−0.0184 (10)	−0.0152 (10)	−0.0050 (10)
C22	0.0521 (9)	0.0604 (11)	0.0626 (11)	−0.0031 (8)	−0.0051 (8)	−0.0048 (9)

### Geometric parameters (Å, º)

**Table:**

O1—C16	1.1972 (17)	C9—H9A	0.9300
O2—C16	1.3596 (17)	C10—C11	1.378 (2)
O2—C15	1.4562 (16)	C10—H10A	0.9300
O3—C13	1.1973 (17)	C11—C12	1.392 (2)
O4—C13	1.3586 (17)	C11—H11A	0.9300
O4—C14	1.4446 (18)	C12—C13	1.488 (2)
C1—C2	1.384 (2)	C14—C15	1.520 (2)
C1—C6	1.3891 (19)	C14—H14A	0.9700
C1—H1A	0.9300	C14—H14B	0.9700
C2—C3	1.380 (2)	C15—C17	1.5003 (19)
C2—H2A	0.9300	C15—H15A	0.9800
C3—C4	1.376 (2)	C17—C18	1.382 (2)
C3—H3A	0.9300	C17—C22	1.383 (2)
C4—C5	1.3916 (19)	C18—C19	1.392 (2)
C4—H4A	0.9300	C18—H18A	0.9300
C5—C6	1.4068 (18)	C19—C20	1.364 (3)
C5—C16	1.4888 (19)	C19—H19A	0.9300
C6—C7	1.4965 (19)	C20—C21	1.363 (3)
C7—C8	1.393 (2)	C20—H20A	0.9300
C7—C12	1.400 (2)	C21—C22	1.379 (2)
C8—C9	1.384 (2)	C21—H21A	0.9300
C8—H8A	0.9300	C22—H22A	0.9300
C9—C10	1.376 (3)		
			
C16—O2—C15	118.08 (11)	C7—C12—C13	119.56 (12)
C13—O4—C14	117.17 (12)	O3—C13—O4	124.98 (14)
C2—C1—C6	121.21 (14)	O3—C13—C12	125.60 (13)
C2—C1—H1A	119.4	O4—C13—C12	109.39 (13)
C6—C1—H1A	119.4	O4—C14—C15	107.18 (13)
C3—C2—C1	120.35 (14)	O4—C14—H14A	110.3
C3—C2—H2A	119.8	C15—C14—H14A	110.3
C1—C2—H2A	119.8	O4—C14—H14B	110.3
C4—C3—C2	119.66 (14)	C15—C14—H14B	110.3
C4—C3—H3A	120.2	H14A—C14—H14B	108.5
C2—C3—H3A	120.2	O2—C15—C17	108.79 (11)
C3—C4—C5	120.49 (14)	O2—C15—C14	105.86 (11)
C3—C4—H4A	119.8	C17—C15—C14	113.15 (13)
C5—C4—H4A	119.8	O2—C15—H15A	109.6
C4—C5—C6	120.37 (13)	C17—C15—H15A	109.6
C4—C5—C16	119.83 (12)	C14—C15—H15A	109.6
C6—C5—C16	119.77 (12)	O1—C16—O2	124.52 (13)
C1—C6—C5	117.92 (13)	O1—C16—C5	125.93 (13)
C1—C6—C7	118.80 (12)	O2—C16—C5	109.50 (12)
C5—C6—C7	123.27 (12)	C18—C17—C22	118.70 (15)
C8—C7—C12	117.99 (13)	C18—C17—C15	120.77 (15)
C8—C7—C6	118.44 (13)	C22—C17—C15	120.53 (14)
C12—C7—C6	123.57 (13)	C17—C18—C19	120.21 (18)
C9—C8—C7	121.17 (16)	C17—C18—H18A	119.9
C9—C8—H8A	119.4	C19—C18—H18A	119.9
C7—C8—H8A	119.4	C20—C19—C18	120.20 (18)
C10—C9—C8	120.13 (16)	C20—C19—H19A	119.9
C10—C9—H9A	119.9	C18—C19—H19A	119.9
C8—C9—H9A	119.9	C21—C20—C19	119.83 (17)
C9—C10—C11	120.04 (15)	C21—C20—H20A	120.1
C9—C10—H10A	120.0	C19—C20—H20A	120.1
C11—C10—H10A	120.0	C20—C21—C22	120.74 (19)
C10—C11—C12	120.17 (16)	C20—C21—H21A	119.6
C10—C11—H11A	119.9	C22—C21—H21A	119.6
C12—C11—H11A	119.9	C21—C22—C17	120.28 (17)
C11—C12—C7	120.49 (14)	C21—C22—H22A	119.9
C11—C12—C13	119.94 (14)	C17—C22—H22A	119.9
			
C6—C1—C2—C3	−0.4 (3)	C11—C12—C13—O3	129.03 (16)
C1—C2—C3—C4	0.8 (3)	C7—C12—C13—O3	−51.7 (2)
C2—C3—C4—C5	−0.6 (3)	C11—C12—C13—O4	−53.03 (17)
C3—C4—C5—C6	0.1 (2)	C7—C12—C13—O4	126.21 (13)
C3—C4—C5—C16	178.33 (15)	C13—O4—C14—C15	92.18 (15)
C2—C1—C6—C5	−0.2 (2)	C16—O2—C15—C17	−141.79 (12)
C2—C1—C6—C7	−179.94 (15)	C16—O2—C15—C14	96.32 (14)
C4—C5—C6—C1	0.3 (2)	O4—C14—C15—O2	−65.31 (15)
C16—C5—C6—C1	−177.94 (14)	O4—C14—C15—C17	175.64 (12)
C4—C5—C6—C7	−179.93 (14)	C15—O2—C16—O1	18.96 (19)
C16—C5—C6—C7	1.8 (2)	C15—O2—C16—C5	−158.71 (11)
C1—C6—C7—C8	−56.42 (19)	C4—C5—C16—O1	124.59 (16)
C5—C6—C7—C8	123.83 (15)	C6—C5—C16—O1	−57.1 (2)
C1—C6—C7—C12	123.92 (16)	C4—C5—C16—O2	−57.78 (17)
C5—C6—C7—C12	−55.8 (2)	C6—C5—C16—O2	120.49 (14)
C12—C7—C8—C9	0.5 (2)	O2—C15—C17—C18	132.03 (15)
C6—C7—C8—C9	−179.19 (14)	C14—C15—C17—C18	−110.62 (17)
C7—C8—C9—C10	0.2 (3)	O2—C15—C17—C22	−48.06 (19)
C8—C9—C10—C11	0.1 (3)	C14—C15—C17—C22	69.29 (19)
C9—C10—C11—C12	−1.0 (2)	C22—C17—C18—C19	0.6 (3)
C10—C11—C12—C7	1.6 (2)	C15—C17—C18—C19	−179.52 (16)
C10—C11—C12—C13	−179.13 (14)	C17—C18—C19—C20	1.3 (3)
C8—C7—C12—C11	−1.4 (2)	C18—C19—C20—C21	−2.2 (3)
C6—C7—C12—C11	178.28 (13)	C19—C20—C21—C22	1.3 (3)
C8—C7—C12—C13	179.39 (13)	C20—C21—C22—C17	0.6 (3)
C6—C7—C12—C13	−1.0 (2)	C18—C17—C22—C21	−1.5 (3)
C14—O4—C13—O3	21.5 (2)	C15—C17—C22—C21	178.61 (16)
C14—O4—C13—C12	−156.50 (12)		
